# A comparative study of PCNA and Ki-67 expression in dental follicle, dentigerous cyst, unicystic ameloblastoma and ameloblastoma

**Published:** 2013

**Authors:** Shima Nafarzadeh, Maryam Seyedmajidi, Sina Jafari, Ali Bijani, Ali Rostami-Sarokolaei

**Affiliations:** 1 *Dental material research center, Babol University of Medical Sciences, Babol, Iran.*; 2 *Department of Oral and Maxillofacial Pathology, Dentistry school, Babol University of Medical Sciences, Babol, Iran.*; 3 *Student Research Committee, Babol University of Medical Sciences, Babol, Iran.*; 4 *Non-communicable Pediatric Diseases Research Center, Babol University of Medical Sciences, Babol, Iran.*; 5 *Dentistry school, Babol University of Medical Sciences, Babol, Iran.*

**Keywords:** PCNA, Ki-67, immunohistochemistry, dental follicle, dentigerous cyst, ameloblastoma

## Abstract

Various cell proliferation markers are used as diagnostic and prognostic tools in oral lesions. Simultaneous evaluation of these markers can increase the precision of estimation of the proliferative status of different tissues.

In this study we investigated the expression of PCNA and Ki-67 as markers of cell proliferation in 15 paraffin embedded samples of each dental follicle, dentigerous cyst, unicystic ameloblastoma and ameloblastoma belonging to a total of 30 male and 30 female paients using immunohistochemistry method. Expression levels based on the intensity and the percentage of stained cells was separately analyzed for each marker with chi-square test, the results of which were significant for the two markers (P<0.05). The correlation coefficient between the two markers was found to be 0.88. A significant difference in the expression of Ki-67 and PCNA was observed in the four types of studied lesions.

Dental follicle originates from odontogenic ectomesenchyme and is one of the components of the tooth germ. It is composed of two parts including the crown and the root which itself includes enamel organ, dental papilla, and dental follicle. The developing tooth germ is surrounded by a dense embryonic connective tissue known as dental sac or dental follicle (1, 2). Dentigerous cyst as the most common developmental odontogenic cyst, accounts for 20% of all epithelium-lined jaw cysts. The cyst encloses an undeveloped crown and attaches to the tooth at cemento-enamel junction (CEJ). Although the pathogenesis of the cyst is unknown, it obviously develops due to fluid accumulation between the reduced enamel epithelium and the tooth crown. The cyst is often found at the age of 10 to 30 years and is more common in males with male to female ratio of 1.6:1 (1, 2). Ameloblastoma is an odontogenic tumor, which accounts for 1% of head and neck tumors. It grows slowly without any pain and symptom, and despite the benign nature of the lesion, localized invasion has been reported. The lesion tends to have high recurrence and metastases in rare cases. Unicystic Ameloblastoma (UA) was first described in 1977 by Robinson and Martinez (3). UA is most commonly observed in the mandibular molar-ramus region. The lesion is in some cases associated with impacted third molars. It is most commonly encountered at the second decade of life. Microscopically, the lesion has three patterns: Luminal, Intraluminal and Mural (1, 2). 

There are different methods for evaluating oral lesions in which certain proteins and other substances in the lesion are directly and indirectly measured (4, 5). The 395-kD protein – Ki-67 – is expressed in proliferating cells and during DNA synthesis and immediately disappears after mitosis (6, 7). This antigen is preferentially expressed during late G1, S, G2 and M phase, whereas resting, non-cycling cells (G0 phase) lack Ki-67 expression. Because of its absence in quiescent cells (G0 phase), this protein developed into a widely used tumor marker in the fields of research and pathology (8). Ki-67 is of prognostic value for many types of malignant tumors (9). Proliferating nuclear cell antigen (PCNA) is known as an important protein in DNA synthesis and repair (6, 7).

This nuclear non-histone protein is an accessory protein for DNA polymerase alpha, an essential factor for DNA replication and repair. This protein is elevated during the G1/S phase (10).

Various cell proliferation markers have been used in several studies as diagnostic and prognostic tools as well as aids in understanding the biological behavior in many stages of disease. Currently, new markers are being added to evaluate cell pro-liferation. However, PCNA is still used as a marker of cell proliferation, and Ki-67 is considered the classic marker of cell proliferation and is in routine use by pathologists. Furthermore, several studies have been performed to evaluate cell proliferation using PCNA and Ki-67 in different tumors of various origins; compared with PCNA, Ki-67 has been shown to be more sensitive and specific in the various tumors analyzed (6-11).

Despite the existence of these data, there are still numerous studies using PCNA as the first-choice marker of cell proliferation. Many investigations of tumor-cell proliferative activity have used PCNA and Ki-67 to evaluate cell proliferation in oral tumors (11-13). It is known that Ki-67 is a more specific marker for the proliferation of ameloblastic tumor cells (4). 

Therefore, it seems that simultaneous evaluation of these two markers can be a precise estimation for the proliferative function of tissues and different tumors that can also be helpful in determining progression, aggressiveness and prognosis of the lesions. Hence, the present study aimed to investigate the expression of Ki-67 and PCNA in dental follicle, dentigerous cyst, unicystic ameloblastoma and ameloblastoma using immuno-histochemistry method.

## Materials and Methods

The present analytical cross-sectional study was conducted on archived samples of Oral and Maxillofacial Pathology department of dental school of Babol University of Medical Sciences during 2004-2009, among which those samples diagnosed as ameloblastoma, unicystic amelo-blastoma, dentigerous cyst and dental follicle were selected. To complete the number of samples, samples of Shahid Beheshti Hospital were also used. A total of 60 paraffin blocks including 15 cases of ameloblastoma, 15 unicystic amelo-blastomas, 15 dentigerous cysts and 15 dental follicles were studied. Patients information including age, gender, and tumor location were extracted from the records. One 4-micron section was obtained from each block using the microtome, stained with Hematoxylin and Eosin (H&E) staining and re-examined for the confirmation of diagnosis. Blocks with adequate amount of tissue were selected, from each, three micron sections were prepared for immunohistochemical studies.

Immunohistochemistry was performed using the standard method (Avidin Biotin Peroxidase)(4, 5). The sections were deparaffinized and then dehydrated. In the next step, blocks were incubated in fresh citrate/HCl buffer solution (Colon PClo) for antigen recovery and were washed one more time with PBS after one hour incubation. Samples were then incubated with biotinylated monoclonal antibodies against Ki-67 (clone MIB-1; 1:100 dilution, Dako, Carpinteria, CA, USA) and PCNA (Clone PC10; dilution 1:100, Dako, Carpinteria, CA, USA). Then the slides were exposed to peroxidase-labeled streptavidin followed by washing with PBS, and were finally exposed to DAB chromogen (Diaminobenzidine hydrochloride). 

Afterwards, the slides were stained with Myers hematoxylin and were covered with coverslips following dehumidification. Stained slides were observed by a pathologist using an Olympus BX41 (Olympus, Tokyo, Japan) light microscope under 40× magnification. For each slide, H-score grading level, the degree of staining intensity and the percentage of stained epithelial cells were considered. The grading system for staining intensity is as follows. Score0 (No staining), Score1 (Weak), Score2 (Moderate), and Score3 (Intense).

The total number of epithelial cells, including negative and positive cells, was counted, and the percentage of positive cells was calculated. Immunolabeling was analyzed semiquantitatively using the following scores according to the percentage of positive cells: Score 1 (<1%), Score2 (1–10%), Score3 (11–33%), Score4 (34-66%), and Score5 (67-100%).

The sum of these two was then considered as the final score (9); data were analyzed by SPSS18 statistical software using Mann-Whitney, Pearson correlation, Chi-Square and One Way ANOVA tests. Quantitative data are presented as mean (± Standard Deviation) and qualitative data as frequency.

## Results

**Fig 1:**
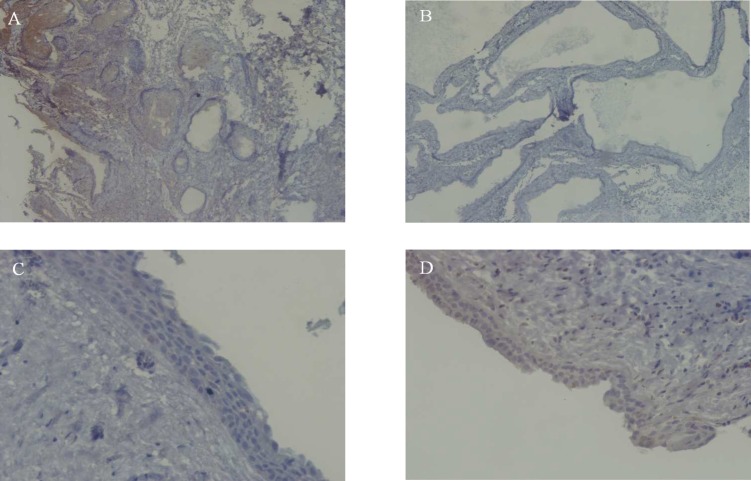
Immunohistochemical staining with Ki-67 in Ameloblastoma, Unicystic ameloblastoma, Dentigerous cyst and Dental follicle. (A,B,C,D respectively

**Fig 2:**
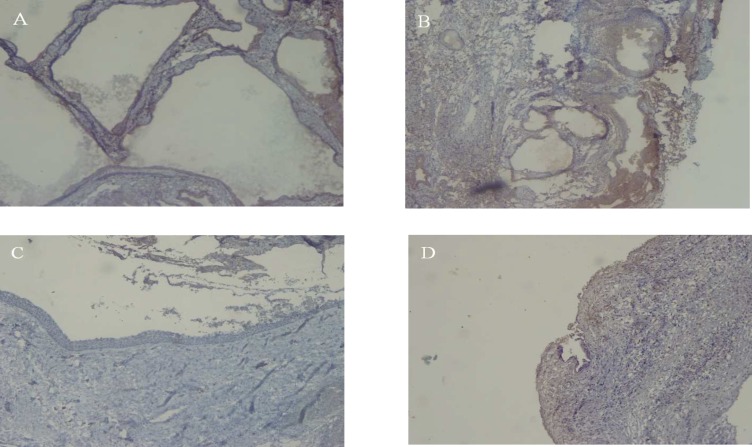
**. **Immunohistochemical staining with Ki-67 in Ameloblastoma, Unicystic ameloblastoma, Dentigerous cyst and Dental follicle. (A,B,C,D respectively

Out of 60 samples studied, 30 patients were male and 30 were female. The mean age of patients was 19.57 (± 11.35) years, ranging from 5 to 59 years. The expression of Ki-67 (Fig 1) and PCNA (Fig 2) in dental follicle, dentigerous cyst, unicystic ameloblastoma and ameloblastoma based on the staining intensity and the percentage of stained cells are presented in Tables 1 and 2 respectively.

The correlation coefficient between the two markers was found to be 0.88, showing a statistically significant correlation (P<0.001). Expression levels of Ki-67 and PCNA based on the intensity of stained cells was separately analyzed with chi-square test, the results of which were significant for the two markers (P<0.05). Expression levels of Ki-67 and PCNA based on the percentage of stained cells was also analyzed separately by chi-square statistical test and was observed to be statistically meaningful for the two markers (P<0.05). 

Total Ki-67 level in different samples has been analyzed by ANOVA statistical test, and the difference in the results obtained showed a significant differences in at least one of the studied types (P<0.001). 

Total PCNA level has also been analyzed in different samples by the mentioned test and the results were statistically significant (P<0.001). In the end, total Ki-67 and total PCNA levels were analyzed separately in the four types of samples and the results were presented in Table 2; As shown, total Ki-67 level revealed no statistical difference only between dentigerous cyst and unicystic ameloblastoma (P>0.05).

**Table 1:** Expression of Ki-67 and PCNA in the dental follicle, dentigerous cyst, unicystic ameloblastoma and ameloblastoma based on the staining intensity

Type of samples	Number of samples		Weak	Moderate	Intense
Dental follicle	15	Ki-67	12	3	0
		PCNA	12	3	0
Dentigerous cyst	15	Ki-67	0	14	1
		PCNA	0	14	1
Unicystic ameloblastoma	15	Ki-67	0	10	5
		PCNA	0	12	3
Ameloblastoma	15	Ki-67	0	5	10
		PCNA	0	2	13

**Table 2:** Expression of Ki-67 and PCNA in the dental follicle, dentigerous cyst, unicystic ameloblastoma and ameloblastoma based on the percentage of stained cells

Type of samples	Number of samples		Score 2	Score 3	Score 4	Score 5	Mean (±SD)
Dental follicle (DF)	15	Ki-67	13	2	0	0	3.26 (±0.59)
		PCNA	5	10	0	0	2.39 (±0.59)
Dentigerous cyst (DC)	15	Ki-67	1	10	3	1	5.33 (±0.72) *
		PCNA	3	11	1	0	5.33 (±0.72)¶
Unicystic ameloblastoma (UA)	15	Ki-67	0	4	10	1	6.13 (±0.83) *
		PCNA	0	9	6	0	5.60 (±0.63)¶ θ
Ameloblastoma (A)	15	Ki-67	0	0	7	8	7.20 (±0.67)* ¥ §
		PCNA	0	0	8	7	7.33 (±0.73)¶ θ η

One Way ANOVA test P-values for Ki-67 (* DF vs. DC, UA, A P<0.001; ¥ DC vs. A P<0.001; § UA vs. A P<0.05), PCNA (¶ DF vs. DC, UA, A P<0.001; θ DC vs. UA, A P<0.001; η UA vs. A P<0.001).

## Discussion

In the present study, dental follicle, dentigerous cyst, unicystic ameloblastoma and ameloblastoma were evaluated in terms of Ki-67 and PCNA expression levels to justify the recurrence and different clinical behaviors.

The results of our study showed a significant difference in the expression of Ki-67 and PCNA in the lesions studied. Moreover, regarding the correlation between Ki-67 and PCNA antibodies (0.88), it has been found that there was a significant relationship between them. Mashhadi et al. showed that increased expression of these two markers could be involved in the pathogenesis of denti-gerous cyst (14). In a research by Meer et al., the expression of these two markers has been studied in ameloblastoma and it has been revealed that the proliferation activity of Ki-67 and PCNA is different in different types of ameloblastoma and the expression level of these two markers is higher in unicystic ameloblastoma compared with multi-cystic ameloblastoma (15). Other studies, such as Shear et al. (16) and Li et al. (17) had pointed to the involvement of Ki-67 and PCNA in the patho-genesis of different lesions and considered the two markers as a mean to justify the recurrence and aggressive behavior of tumors. In a study by Bologna-Molina et al. on Ki-67 expression in 120 samples of ameloblastoma, it has been demo-nstrated that Ki-67 expression is similar in multi- and unicystic ameloblastoma, among which there is no significant difference (18). Güler et al. showed that Ki-67 and MCM-2 markers expression were statistically significant in mucous cell prosoplasia and squamous epithelium of dental follicles where Ki-67 expressions were more significant in glandular epithelium of dental follicle (19). Razavi et al. reported that Ki-67 expression was significantly higher in solid ameloblastoma compared to adenomatoid odontogenic tumor (20). In the most of the mentioned studies, the expression level of markers has been evaluated only in two types of lesions; however, four types of lesions have been evaluated in the present research. Since the procedures in immunohistochemical studies affect the results, the reason of some differences between the results of the present study and other investigations are probably due to different experimental conditions such as appropriate pH for citrate buffer, duration of slice exposure to hydrogen peroxide solution, hydrogen peroxide concentration and the type of antibody used. Also, the difference in appropriate cut-of-point in various studies affects the interpretation of results. In the process of cell proliferation, there is a need for cell division under the control of molecules expressed during the cell cycle (Ki-67 and PCNA). Imbalance or increase of cell proliferation have been reported in various lesions such as tumors and cysts and it is expected that in lesions with higher levels of invasion, the amount of molecules involved in cell cycle be different to those with less invasion.
